# Characteristics of Patients With Cancer and COVID-19 Who Discontinued Cancer Treatment

**DOI:** 10.1001/jamanetworkopen.2024.11859

**Published:** 2024-05-23

**Authors:** Jessica Y. Islam, Cassandra A. Hathaway, Emma Hume, Kea Turner, Julie Hallanger-Johnson, Shelley S. Tworoger, Marlene Camacho-Rivera

**Affiliations:** 1 Center for Immunization and Infection Research in Cancer, H. Lee Moffitt Cancer Center and Research Institute, Tampa, Florida; 2 Department of Cancer Epidemiology, H. Lee Moffitt Cancer Center and Research Institute, Tampa, Florida; 3 Department of Health Outcomes and Behavior, H. Lee Moffitt Cancer Center and Research Institute, Tampa, Florida; 4 Department of Gastrointestinal Oncology, H. Lee Moffitt Cancer Center and Research Institute, Tampa, Florida; 5 Department of Endocrinology, Mayo Clinic, Rochester, Minnesota; 6 Division of Oncological Sciences, School of Medicine, Oregon Health and Science University, Portland; 7 Department of Community Health Sciences, School of Public Health, SUNY Downstate Health Sciences University, New York, New York

## Abstract

This cross-sectional study evaluates the prevalence of and characteristics associated with discontinuation of cancer treatment among patients who received a diagnosis of COVID-19 during their treatment planning.

## Introduction

The COVID-19 pandemic disrupted access to cancer treatment.^1^ COVID-19–related disruptions in treatment may have disproportionately affected individuals who experienced a high burden of COVID-19–related disease.^1^ Our objective was to evaluate cancer treatment discontinuations (TDs) among patients with cancer who received a diagnosis of SARS-CoV-2 during their cancer treatment planning.

## Methods

Data for this cross-sectional study were obtained from the American Society of Clinical Oncology Survey on COVID-19 in Oncology Registry^2^ (March 2020 to September 2022). Participating clinics included patients according to 2 eligibility criteria: (1) confirmed SARS-CoV-2 infection and (2) engagement in care at the time of infection. TD was defined as discontinued or canceled treatment with no plans of restart as reported by the clinic. Median (IQR) time between COVID-19 diagnosis date and the date TD status was ascertained was 2 (0-5) months. Owing to the descriptive nature of this analysis, we did not include an adjustment for multiple comparisons.^3^ The study was reviewed by the Scientific Review Committee at Moffitt Cancer Center and was deemed as nonhuman participants research. The study was reported in accordance with the STROBE reporting guidelines.^4^ Further details regarding the study methods have been previously published^2^ and are shown in Supplement 1.

## Results

The study population included 3812 patients (3125 patients [82%] aged ≥50 years; 2286 female [60%]; 2595 non-Hispanic White patients [68%]; 443 Hispanic patients [12%]; and 3290 patients living with ≤2 comorbidities [86%]) (Table). Overall, the prevalence of TD was 5.3% (200 of 3182 patients), with 3454 (90.6%) scheduled for drug-based therapy, 361 (9.5%) for radiation, 216 (5.7%) for surgery, and 30 (0.8%) transplant or cellular therapy. Controlling for other factors, non-Hispanic Asian patients had a much higher likelihood of experiencing cancer TD vs their non-Hispanic White counterparts (adjusted prevalence ratio [aPR], 1.62; 95% CI, 1.13-2.32) (Figure). Clinical factors associated with elevated likelihood of cancer TD included a higher (≥3) Eastern Cooperative Oncology Group score (adjusted prevalence ratio [aPR], 2.61; 95% CI, 1.12-4.36) and diagnosis with a gastrointestinal cancer (aPR, 2.13; 95% CI, 1.29-3.52). Severe COVID-19 outcomes were associated with a higher likelihood of cancer TD. Patients with stable cancer at COVID-19 diagnosis were less likely to experience cancer TD compared with those with progressing cancer (aPR, 0.51; 95% CI, 0.36-0.71).

**Table.** Characteristics of Clinics and Patients With Cancer and SARS-CoV-2 Infection During Treatment Planning Captured in the ASCO Cancer and COVID-19 Registry Stratified by Treatment Discontinuation

Characteristic	Total, No. (%) (N = 3812)^a^	Treatment cancellations and discontinuations without plans to restart treatment, No. (%)^b^		*P* value
		No treatment impact	Treatment impacted	
Clinic characteristics^c^				
Practice type				
Academic institution	16 (23.2)	NA	NA	NA
Nonacademic hospital or health system	37 (53.6)	NA	NA	
Physician-owned, independent practice	16 (23.2)	NA	NA	
Census region				
Midwest	22 (31.9)	NA	NA	NA
Northeast	12 (17.4)	NA	NA	
South	25 (36.2)	NA	NA	
West	10 (14.5)	NA	NA	
Patient sociodemographics				
Age category at SARS-CoV-2 infection diagnosis, y^c^				
18-34	139 (3.7)	131 (94.2)	8 (5.8)	.02
35-49	533 (14.0)	509 (95.5)	24 (4.5)	
50-64	1359 (35.8)	1305 (96.0)	54 (4.0)	
≥65	1766 (46.5)	1652 (93.5)	114 (6.5)	
Missing	15 (0.4)	15 (100)	0	
COVID-19 case surge waves				
First wave (March-June 2020)	157 (4.1)	128 (81.5)	29 (18.5)	<.001
Second wave (July-November 2020)	749 (19.6)	680 (90.8)	69 (9.2)	
Third wave (December 2020-March 2021)	983(25.8)	941 (95.7)	42 (4.3)	
Fourth wave (April 2021-February 2022)	1317 (34.5)	1274 (96.7)	43 (3.3)	
Fifth wave (March-September 2022)	606 (15.9)	589 (97.2)	17 (2.8)	
Sex				
Male	1526 (40.0)	1440 (94.4)	86 (5.6)	.38
Female	2286 (60.0)	2172 (95.0)	114 (5.0)	
Race and ethnicity				
Hispanic	443 (11.6)	420 (94.8)	23 (5.2)	
Non-Hispanic American Indian or Alaska Native	134 (3.5)	130 (97.0)	4 (3.0)	
Non-Hispanic Asian	162 (4.3)	146 (90.1)	16 (9.9)	
Non-Hispanic Black	477 (12.5)	440 (92.2)	37 (7.8)	
Non-Hispanic White	2595 (68.1)	2475 (95.4)	120 (4.6)	.002
Missing	1 (0)	1 (100)	0 (0)	
Rurality of patient’s residence				
Urban	3228 (84.7)	3048 (94.4)	180 (5.6)	.03
Rural	583 (15.3)	563 (96.6)	20 (3.4)	
Missing	1 (0.0)	1 (100)	0	
Census region				
Midwest	1219 (32.0)	1166 (95.7)	53 (4.3)	<.001
Northeast	490 (12.9)	443 (90.4)	47 (9.6)	
South	1787 (46.9)	1717 (96.1)	70 (3.9)	
West	315 (8.3)	285 (90.5)	30 (9.5)	
Missing	1 (0.0)	1 (100)	0	
No. of comorbidities				
None	1456 (38.2)	1411 (96.9)	45 (3.1)	<.001
1-2	1834 (48.1)	1734 (94.5)	100 (5.5)	
3-4	388 (10.2)	353 (91.0)	35 (9.0)	
≥5	134 (3.5)	114 (85.1)	20 (14.9)	
Clinical cancer characteristics				
Cancer type				
Breast	1078 (28.3)	1046 (97.0)	32 (3.0)	<.001
Lung	374 (9.8)	347 (92.8)	27 (7.2)	
Genitourinary	332 (8.7)	319 (96.1)	13 (3.9)	
Gastrointestinal	450 (11.8)	407 (90.4)	43 (9.6)	
Gynecological	216 (5.7)	197 (91.2)	19 (8.8)	
Leukemia, lymphomas, and myeloma	879 (23.1)	843 (95.9)	36 (4.1)	
Other solid tumors	483 (12.7)	453 (93.8)	30 (6.2)	
Cancer diagnosis year				
2010 or earlier	202 (5.3)	195 (96.5)	7 (3.5)	.34
2011-2019	1738 (45.6)	1639 (94.3)	99 (5.7)	
2020-2022	1870 (49.1)	1776 (95.0)	94 (5.0)	
Missing	2 (0.1)	2 (100)	0	
Eastern Cooperative Oncology Group status^c^				
0	1286 (42.5)	1256 (97.7)	30 (2.3)	<.001
1	1222 (40.4)	1173 (96.0)	49 (4.0)	
2	382 (12.6)	346 (90.6)	36 (9.4)	
≥3	134 (4.4)	105 (78.4)	29 (21.6)	
Missing	788 (20.7)	732 (92.9)	56 (7.1)	
Tobacco use				
Current smoker	352 (9.2)	338 (96.0)	14 (4.0)	.11
Former smoker	1429 (37.5)	1339 (93.7)	90 (6.3)	
Never smoked	1918 (50.3)	1829 (95.4)	89 (4.6)	
Unsure	113 (3.0)	106 (93.8)	7 (6.2)	
Body mass index^d^				
Underweight (<18.5)	84 (2.3)	70 (83.3)	14 (16.7)	<.001
Healthy weight (18.5 to <25.0)	952 (25.7)	899 (94.4)	53 (5.6)	
Overweight (25.0 to <30.0)	1181 (31.9)	1114 (94.3)	67 (5.7)	
Obesity (≥30.0)	1491 (40.2)	1436 (96.3)	55 (3.7)	
Missing	104 (2.7)	93 (89.4)	11 (10.6)	
Extent of cancer^c^				
Local	811 (21.3)	775 (95.6)	36 (4.4)	.01
Regional	408 (10.7)	389 (95.3)	19 (4.7)	
Metastatic	1396 (36.6)	1300 (93.1)	96 (6.9)	
Cancer-free but receiving adjuvant therapy	222 (5.8)	215 (96.8)	7 (3.2)	
Missing or unknown	975 (25.6)	933 (95.7)	42 (4.3)	
Cancer status at the time of COVID-19 diagnosis				
Progressing	481 (12.6)	424 (88.1)	57 (11.9)	<.001
Stable	1215 (31.9)	1167 (96.0)	48 (4.0)	
Responding to treatment	267 (7.0)	257 (96.3)	10 (3.7)	
Missing or unknown	1849 (48.5)	1764 (190.1)	85 (9.9)	
Surgery to resect or remove cancer within 6 wk before COVID-19 diagnosis				
No	3545 (93.0)	3356 (94.7)	189 (5.3)	.41
Yes	92 (2.4)	90 (97.8)	2 (2.2)	
Unknown	175 (4.6)	166 (94.9)	9 (5.1)	
Scheduled treatment at the time of COVID-19 diagnosis				
Surgery scheduled within 0-6 wk after COVID-19 diagnosis	216 (5.7)	211 (97.7)	5 (2.3)	.05
Radiation therapy	361 (9.5)	329 (91.1)	32 (8.9)	.001
Drug-based therapy	3454 (90.6)	3267 (94.6)	187 (5.4)	.15
Transplant	30 (0.8)	29 (96.7)	1 (3.3)	.64
Patient COVID-19 information				
Patient vaccinated for COVID-19 (before or after COVID-19 diagnosis)				
No	911 (35.5)	870 (95.5)	41 (4.5)	.01
Yes	1019 (39.8)	985 (96.7)	34 (3.3)	
Unsure	633 (24.7)	623 (98.4)	10 (1.6)	
COVID-19 diagnosis severity				
Uncomplicated	2784 (73.0)	2716 (97.6)	68 (2.4)	<.001
Hospitalized	892 (23.4)	793 (88.9)	99 (11.1)	
Intensive care unit admission	90 (2.4)	78 (86.7)	12 (13.3)	
Mechanically ventilated	46 (1.2)	25 (54.3)	21 (45.7)	

Abbreviations: ASCO, American Society of Clinical Oncology; NA, not applicable.

^a^ Denotes column percentage.

^b^ Denotes row percentage.

^c^ Data were provided from the ASCO Registry at the aggregate level.

^d^ Body mass index is calculated as weight in kilograms divided by height in meters squared.

**Figure.**
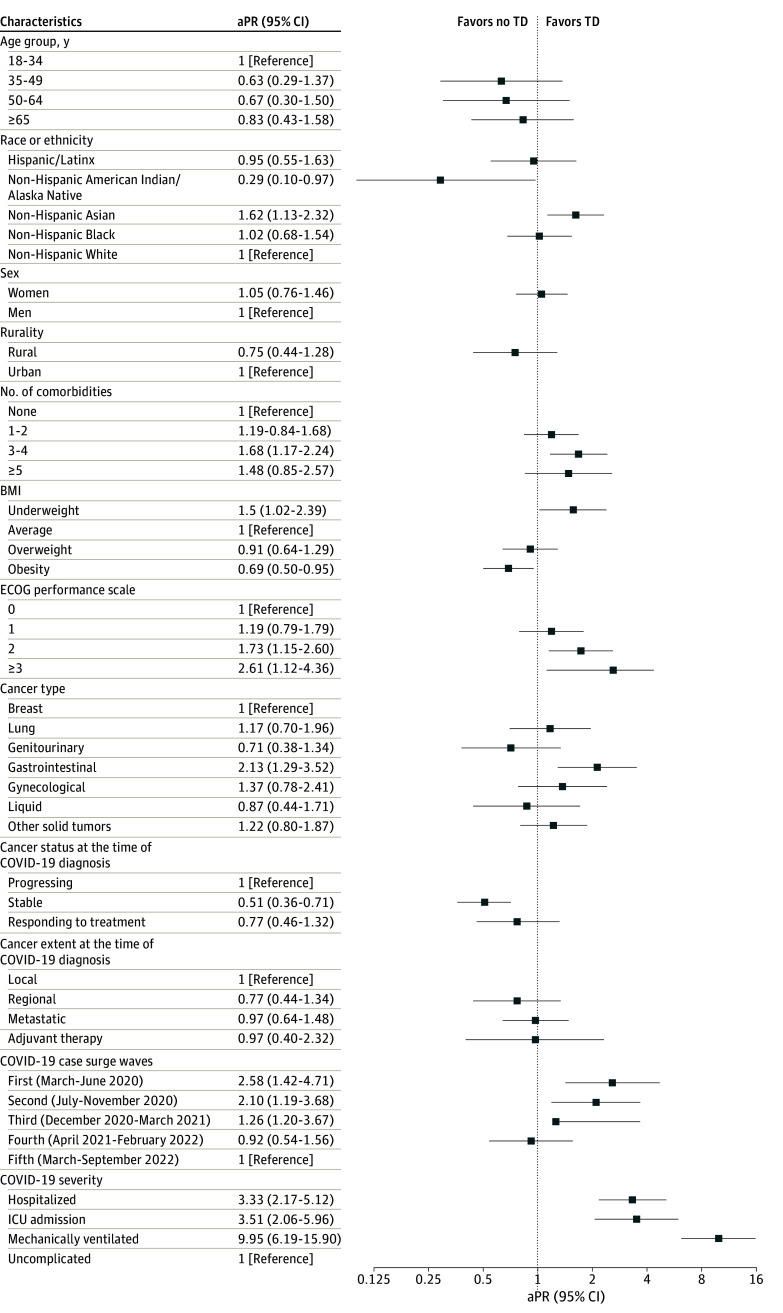
Associations of Sociodemographic and Clinical Factors With Cancer Treatment Discontinuations (TDs) Among Patients With Cancer With SARS-CoV-2 Infection Diagnosed During Cancer Treatment Planning Data are from the American Society of Clinical Oncology Survey on COVID-19 in Oncology Registry (3812 patients). aPR indicates adjusted prevalence ratio; BMI, body mass index; ECOG, Eastern Cooperative Oncology Group; ICU, intensive care unit.

## Discussion

To our knowledge, this cross-sectional study of cancer treatment TD is novel because we focus on a population of patients with cancer who experienced severe interruptions in care during the COVID-19 pandemic. Importantly, TD was more likely to occur during the early phases of the pandemic, which may be due to multiple reasons such as availability of COVID-19 vaccination, and changes in response of health care systems once safety protocols were established.^1^ We found that non-Hispanic Asian patients with cancer were most likely to experience TD. Multiple factors may have led to this racial inequity in receipt of cancer treatment, including difficulties leveraging telehealth due to language barriers among older Asian patients and the higher likelihood of hospitalization and death due to COVID-19 compared with White adults.^5^ Clinical factors identified pertinent to cancer TD, such as gastrointestinal cancer diagnosis or severe COVID-19 disease, is similar to prior analysis that focused on any cancer treatment delays of at least 14 days.^6^ Of note, cancer TD was less likely to occur among patients with stable cancer vs those with progressing cancers. This may suggest that patients already engaged in care were able to continue treatment. An important limitation of our data is that the type of cancer treatment (ie, adjuvant, palliative) was not available, which may have affected the risk or benefit for completing treatment. Nonetheless, our findings suggest long-term consequences of cancer treatment cancellations should be monitored to identify any potential exacerbations in inequities in cancer outcomes due to the COVID-19 pandemic.

## Notes

Supplement 1. **eAppendix.** Supplemental Methods

Supplement 2.
Data Sharing Statement
