# Supplementary material for: Characteristics of Patients With Cancer and COVID-19 Who Discontinued Cancer Treatment
Source: JAMA Netw Open. 2024 May 23;7(5):e2411859. doi: 10.1001/jamanetworkopen.2024.11859 (PMC11117082; doi:10.1001/jamanetworkopen.2024.11859)
Supplement: Supplement 1. — eAppendix. Supplemental Methods [file jamanetwopen-e2411859-s001.pdf]

Supplemental Online Content

Islam JY, Hathaway CA, Hume E, et al. Characteristics of patients with cancer and COVID-19 who discontinued cancer treatment. *JAMA Netw Open*. 2024;7(5):e2411859. doi:10.1001/jamanetworkopen.2024.11859

eAppendix. Supplemental Methods

This supplemental material has been provided by the authors to give readers additional information about their work.

© 2024 Islam JY et al. *JAMA Network Open.*

eAppendix. Supplemental Methods

We descriptively evaluated demographic and clinical characteristics by TD status. We estimated adjusted prevalence ratios (aPRs) using multivariable Poisson regression using robust estimation of SEs, accounting for nonindependence of patients within hospitals. Multivariable models included age, self-reported race or ethnicity, sex, number of comorbidities, body mass index (calculated as weight in kilograms divided by height in meters squared), cancer type, cancer extent, cancer status at SARS-CoV-2 diagnosis (progressing or stable), performance status or Eastern Cooperative Oncology Group (ECOG) score, COVID-19 severity (uncomplicated, hospitalization, intensive care unit admission, or mechanical ventilation), and COVID-19 case surge wave periods. Race and ethnicity (Hispanic/Latinx, non-Hispanic [NH] American Indian or Alaska Native, non-Hispanic Asian, non-Hispanic Black, and non-Hispanic White) were collected by the ASCO registry as sociodemographic information for each patient. Collinearity was assessed using the variance inflation factor. Patients who died within 30 days due to their COVID-19 disease were excluded.

Tests were 2-tailed, and statistical significance was set at $P < .05$. Data were analyzed from September to December 2023.

© 2024 Islam JY et al. *JAMA Network Open.*
