# Supplementary material for: Characteristics of Patients With Cancer and COVID-19 Who Discontinued Cancer Treatment
Source: JAMA Netw Open. 2024 May 23;7(5):e2411859. doi: 10.1001/jamanetworkopen.2024.11859 (PMC11117082; doi:10.1001/jamanetworkopen.2024.11859)
Supplement: Supplement 2. — Data Sharing Statement [file jamanetwopen-e2411859-s002.pdf]

Data Sharing Statement

Islam. Characteristics of Patients With Cancer and COVID-19 Who Discontinued Cancer Treatment. *JAMA Netw Open*. Published May 17, 2024.
doi:10.1001/jamanetworkopen.2024.11859

Data
Data available: No

Additional Information
Explanation for why data not available: Data can be requested from the American Society of Clinical Oncology (ASCO) Cancer and COVID-19 registry team.
